# Abnormal Brain Activation During Verbal Memory Encoding in Postacute Anti-N-Methyl-d-Aspartate Receptor Encephalitis

**DOI:** 10.1089/brain.2021.0046

**Published:** 2022-09-15

**Authors:** Kang Wang, Dengchang Wu, Caihong Ji, Benyan Luo, Chunjie Wang, Zhongqin Chen

**Affiliations:** ^1^ Department of Neurology, The First Affiliated Hospital, School of Medicine, Zhejiang University, Hangzhou, China.; ^2^ Institute of Brain Science and Department of Physiology, School of Basic Medical Sciences, Hangzhou Normal University, Hangzhou, China.

**Keywords:** anti-NMDA receptor encephalitis, brain activation, fMRI, hippocampus, verbal memory encoding

## Abstract

**Background:**

Patients with postacute anti-N-methyl-D-aspartate (anti-NMDA) receptor encephalitis are often left with permanent memory impairments. Given that NMDA receptors are essential to memory encoding, and encoding processes have been suggested to contribute to the success of memory retrieval, we investigate whether postacute anti-NMDA receptor encephalitis leads to abnormal brain activation during verbal memory encoding and its potential effects on subsequent memory retrieval performance.

**Methods:**

To address this issue, this study recruited 21 adult patients with anti-NMDA receptor encephalitis past the acute stage and 22 healthy controls (HCs). Functional magnetic resonance imaging (fMRI) data were collected when they completed an episodic memory task.

**Results:**

At the neural level, the patients showed higher brain activation than the HCs in the bilateral hippocampus/parahippocampus (HG/PHG), right superior temporal gyrus (STG), and right thalamus during memory encoding. At the behavioral level, the patients showed worse memory retrieval performance than the HCs. Importantly, greater brain activation in the left HG/PHG during memory encoding was significantly associated with worse memory retrieval performance among the patients.

**Conclusion:**

Our findings indicate that postacute anti-NMDA receptor encephalitis is likely related to altered brain activation during memory encoding. Particularly, less memory retrieval performance often observed in patients with postacute anti-NMDA receptor encephalitis may result from abnormal activation in HG during encoding. These observations may enhance our understanding of NMDA receptor dysfunction in the human brain.

**Impact statement:**

Patients with anti-N-methyl-D-aspartate (anti-NMDA) receptor encephalitis are often left with permanent memory impairments. In this study, brain activation during verbal memory encoding and its potential effects on subsequent memory retrieval performance are addressed using 21 adult patients with postacute anti-NMDA receptor encephalitis and 22 healthy controls. Greater brain activation in the left hippocampus/parahippocampus during memory encoding was significantly associated with worse memory retrieval performance among the patients. These observations enhance our understanding of NMDA receptor dysfunction in the human brain.

## Introduction

Anti-N-methyl-D-aspartate (anti-NMDA) receptor encephalitis is a potentially lethal, but still reversible with treatment, immune-mediated brain disorder associated with IgG antibodies against the NR1 subunit of the NMDA receptor (Dalmau et al., 2008). Generally, timely treatment can lead to substantial recovery, as reflected by low modified Rankin Scale (mRS) scores for ∼80% of patients (Titulaer et al., 2013). However, more than 75% of patients with anti-NMDA receptor encephalitis are left with permanent cognitive deficits of varying severity, especially memory impairments, which become major determinants of long-term morbidity (McKeon et al., 2018). Exploring the neurophysiological mechanisms underlying memory deficits by using multimodal functional magnetic resonance imaging (fMRI) has attracted intensive attention in recent years (Caciagli et al., 2019; Sala-Llonch et al., 2010; Xie et al., 2015). However, to our knowledge, fMRI studies about anti-NMDA receptor encephalitis are very few in literature, which has limited our understanding of this disease.

Importantly, several recent resting-state fMRI studies by our group (Wang et al., 2019) and others (Finke et al., 2013; Peer et al., 2017) have reported that patients with anti-NMDA encephalitis after the acute stage (at least 6 months after initial discharge from the hospital) are usually accompanied by reduced functional connectivity (FC) in the hippocampus (HG). In addition, by the utilization of structural MRI, significantly reduced hippocampal volumes, bilateral atrophy of the input and output regions of the hippocampal circuit, and impaired microstructural integrity of the bilateral HG have been observed in patients with this disease (Finke et al., 2016). Moreover, variations in hippocampal-related FC, hippocampal volumetric, and microstructural integrity measures have been also reported to be significantly correlated with individual memory performance in this population (Finke et al., 2013, 2016; Peer et al., 2017; Wang et al., 2019). Hence, the brain functional and structural abnormalities in the HG may explain some of the memory impairments in anti-NMDAR encephalitis.

Although these studies mentioned above were informative regarding the neural bases underlying memory impairments in patients with anti-NMDA receptor encephalitis, it should be noted that these studies based on resting-state fMRI and structural MRI have simply focused on relationships between brain measures and memory performance on tasks performed outside the MRI scanner. It does not directly explore the neural substrates supporting memory processes in this population. For instance, it remains unknown whether patients with anti-NMDA receptor encephalitis activate different brain regions during memory processes relative to their peers or if they activate the same brain regions, but to a different extent.

In addition, memory comprises multiple processes such as memory encoding, memory consolidation, memory storage, and memory retrieval. Previous studies about anti-NMDA receptor encephalitis have mainly focused on outcomes at memory retrieval processes (Finke et al., 2013, 2016; Peer et al., 2017; Wang et al., 2019). Notably, previous pharmacological, computational, and molecular-genetic studies in rodents have attested to the importance of hippocampal NMDA receptors in mediating memory encoding processes (Morris, 2013; Yamada et al., 2017). In addition, several behavioral studies in humans have shown that encoding new information may be predominately disrupted under the influence of ketamine, an NMDA receptor antagonist with psychotogenic and cognitive effects (Hetem et al., 2000; Honey et al., 2005). Considering these critical impacts of NMDA receptors on memory encoding, it is possible that patients with anti-NMDA receptor encephalitis are accompanied by altered brain activation during memory encoding. However, few studies have directly examined the brain activation patterns during memory encoding in this population, which may provide new insights into the neurophysiological mechanisms of anti-NMDA receptor encephalitis.

The present study thus sets out to determine whether patients with anti-NMDA receptor encephalitis exhibit differential brain activations during memory encoding, compared with healthy controls (HCs). To address this issue, task-based fMRI data were collected when patients with anti-NMDA receptor encephalitis and HC participants completed an episodic memory encoding task. Given that NMDA receptors are essential to memory encoding (Hetem et al., 2000; Honey et al., 2005; Morris, 2013; Yamada et al., 2017), and memory encoding has been suggested to contribute to the success of memory retrieval, we speculated that anti-NMDA receptor encephalitis would lead to altered brain activation during memory encoding, and the altered brain activation might be associated with subsequent memory retrieval impairments.

The HG is one of the medial temporal lobe regions known to be critical for memory encoding (Moscovitch et al., 2016). Based on previous work (Finke et al., 2013, 2016; Peer et al., 2017; Wang et al., 2019) that showed functional and structural differences in hippocampal regions and relationships with memory performance in patients with anti-NMDA receptor encephalitis, we further hypothesized that hippocampal activation may differ between patients with anti-NMDA receptor encephalitis and their peers, and variations in hippocampal activation might be correlated with subsequent memory retrieval deficits in patients with anti-NMDA receptor encephalitis.

## Methods

### Participants

Twenty-one patients with anti-NMDA receptor encephalitis were recruited and had been hospitalized or referred to the outpatient clinic for further counseling and treatment in the Department of Neurology in the First Affiliated Hospital, Zhejiang University School of Medicine. Demographic and clinical data of the patients are shown in Table 1. The diagnosis was based on typical clinical features together with the presence of IgG antibodies for NMDA receptors in the cerebrospinal fluid (CSF) (Dalmau et al., 2011; Graus et al., 2016). Assays for CSF NMDAR-IgG were performed at the EUROIMMUN Diagnostic Laboratory, China, by a cell-based indirect immunofluorescence test (IIFT) using BIOCHIPs (EUROIMMUN AG, Lübeck, Germany).

**Table 1.** Demographic and Clinical Data of the Patients

Patient	IgG NMDA receptor antibodies (CSF titer)	Symptoms		Duration of unconsciousness, days	Treatment protocol	Hospital stay, months	Paraneoplastic syndrome		mRS score		AEDs at study time point	Time between initial discharge and data acquisition, months
		Initial	Total				Tumor	Resection	Before treatment	At study time point		
1	1:32	Behavior, seizure	LOC, dyskinesia, seizure, behavior, cognition	15–20	High-dose steroids, IVIG, cyclophosphamide	1.1	None	/	5	0	Topiramate 50 mg/day	6.5
2	1:32	Behavior, dyskinesia	LOC, dyskinesia, seizure, behavior, cognition, autonomic instability	20–25	High-dose steroids, IVIG, cyclophosphamide	1.2	None	/	5	1	None	9.3
3	1:32	Seizure, LOC	LOC, dyskinesia, seizure, behavior, cognition, autonomic instability	25–30	IVIG, cyclophosphamide	1.4	None	/	5	0	None	21
4	1:32	Dyskinesia, seizure	Dyskinesia, seizure, behavior, cognition	NA	High-dose steroids, IVIG, rituximab	1.2	None	/	4	1	Oxcarbazepine 900 mg/day	6.1
5	1:32	Behavior	LOC, dyskinesia, seizure, behavior, cognition	10–15	High-dose steroids, IVIG, cyclophosphamide	1.2	None	/	5	0	None	18.5
6	1:32	Behavior, cognition	LOC, dyskinesia, seizures, behavior, cognition, autonomic instability	700+	High-dose steroids, plasmapheresis, IVIG, rituximab, cyclophosphamide	26	Mature cystic teratoma of left ovarian	Yes	5	0	None	36.2
7	1:32	Behavior	LOC, dyskinesia, seizures, behavior, cognition	10–15	High-dose steroids, IVIG, cyclophosphamide	0.8	None	/	4	0	None	23.9
8	1:32	Behavior, seizure	LOC, dyskinesia, seizures, behavior, cognition	20–25	High-dose steroids, IVIG, rituximab, cyclophosphamide	1.2	Mature cystic teratoma of bilateral ovarian	Yes	5	0	None	8.3
9	1:3.2	Behavior, dyskinesia	LOC, dyskinesia, seizures, behavior, cognition	20–25	High-dose steroids, IVIG, rituximab, cyclophosphamide	1.3	None	/	5	2	None	13
10	1:3.2	Behavior	LOC, behavior, cognition	8–10	High-dose steroids, IVIG	0.7	None	/	4	0	None	6.5
11	1:10	Seizure, behavior	LOC, dyskinesia, seizure, behavior, cognition	15–20	High-dose steroids, IVIG	1.2	None	/	5	1	None	9.8
12	1:10	Seizure, behavior,	LOC, seizure, behavior, cognition, autonomic instability	20–25	High-dose steroids, IVIG	1.2	None	/	5	1	None	16
13	1:32	Seizure, behavior	Seizure, behavior, cognition	NA	High-dose steroids, IVIG	1.3	Mature cystic teratoma of right ovarian	Yes	5	0	None	23.2
14	1:32	Behavior, seizure	LOC, seizure, behavior, cognition	20–25	High-dose steroids, IVIG	1.3	None	/	4	0	None	21.5
15	1:10	Seizure	LOC, seizure, behavior, cognition	25–30	High-dose steroids, IVIG, rituximab	1.4	None	/	5	1	None	9.1
16	1:32	Behavior	Dyskinesia, seizure, behavior, cognition	NA	High-dose steroids, IVIG, rituximab	1.3	None	/	4	0	None	6.7
17	1:32	Behavior, LOC	LOC, dyskinesia, seizure, behavior, cognition	20–25	High-dose steroids, IVIG, rituximab	0.9	None		5	1	None	13.3
18	1:10	Seizure, behavior	LOC, seizure, behavior, cognition, autonomic instability	25–30	High-dose steroids, IVIG, rituximab	1.4	None	/	5	1	None	9.2
19	1:10	Behavior	Seizure, behavior, cognition	NA	High-dose steroids, IVIG, rituximab	1.2	None	/	4	0	None	10.6

AEDs, antiepileptic drugs; CSF, cerebrospinal fluid; IVIG, intravenous immunoglobulin; LOC, loss of consciousness; mRS, modified Rankin Scale; NA, not applicable; NMDA, N-methyl-D-aspartate.

All patients received first-line immunotherapy (steroids, intravenous immunoglobulin, plasmapheresis) either alone or combined with second-line immunotherapy (rituximab, cyclophosphamide) during the acute phase, according to the clinical treatment guidelines. Although there were differences in the duration of unconsciousness and hospital stay among the patients during the acute phase, all patients achieved good outcome (mRS score, 0–2) at the study time point (Table 1). The fMRI scan was conducted after the acute stage of the disease for all patients (at least 6 months after initial discharge from the hospital).

Twenty-two healthy individuals were recruited as HCs in the experiment. None of the participants presented obvious lesions on routine cranial MRI, previous psychological disorders or seizures, or genetic neurological illness. Two patients and four healthy subjects were excluded because of incomplete fMRI data, failure during behavioral recording, or excessive head motion inside the scanner. As a result, the patients consisted of 19 participants (14 females; mean age = 25.42 years, standard deviation [SD] = 8.69), and the controls included 18 participants (13 females; mean age = 27.61 years, SD = 7.48). The two groups were matched on age [*t*(35) = 0.819, *p* = 0.418] and gender distribution [χ^2^(1,27) = 0.010, *p* = 0.920].

All subjects provided written informed consent, and the project was approved by the Ethics Committee of the First Affiliated Hospital, College of Medicine, Zhejiang University. All the procedures followed were in accordance with the Declaration of Helsinki.

### Episodic memory task

The episodic memory task was adapted from the study of Dhanjal et al. (2013). Stimuli were presented with E-Prime software. Auditory output was recorded using an MR compatible microphone attached to ear defending headphones (MR Confon GmbH, Magdeburg, Germany) to assess performance. A sparse temporal sampling design was used to enable the presentation of auditory stimuli without background auditory interference from the scanner noise (Hall et al., 1999) and to allow short passages of speech production without movement-related artifacts (Dhanjal et al., 2008).

Participants first heard a single episodic sentence (6 sec of memory encoding), in which they were required to remember for subsequent retrieval. The episodic sentences were selected from a children's encyclopedia, a source of simple, highly “imaginable” information, such as “more than half the people in the world eat rice every day,” and recorded by a professional male announcer. Each sentence contained a mean of 6.46 content words (range = 5–8) and a mean of 15.43 unique words (range = 13–17).

**FIG. 1.**
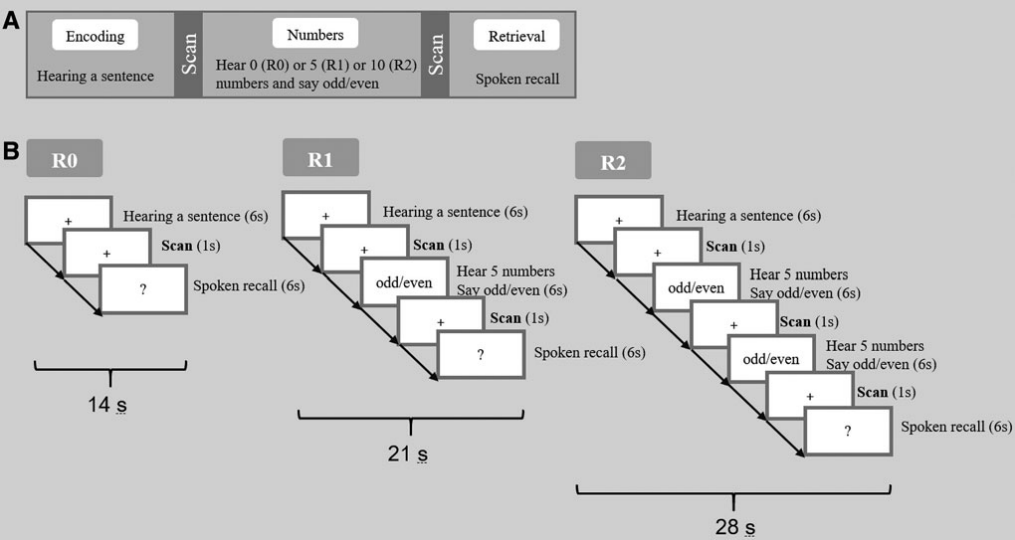
Experimental design of the verbal memory task. **(A)** Event-related design for the fMRI. In each trial, subjects first heard a single sentence (encoding), followed by scanning. Then was followed by a number task (R0: 0 number; or R1: 5 numbers; or R2: 10 numbers), which was pseudorandomly displayed. This was followed by scanning and then followed by spoken free recall of the sentence (retrieval). **(B)** Three task conditions. fMRI, functional magnetic resonance imaging.

Following the memory encoding trial, there were zero, one, or two odd/even number trials (R0, R1, R2). For each number trial, five single numbers were aurally presented at a rate of one number every 1.2 sec, and the participants were required to report whether the current number was odd or even. The odd/even number trial was designed for two purposes. First, it prevented subvocal rehearsal of the previously heard sentence within auditory-verbal working memory before recall. Second, this task could serve as an effective baseline control condition in memory studies since memory-related brain activation has been shown to be uncompromised in such cognitive subtractions (Stark and Squire, 2001). Number trials were followed by a memory retrieval trial (6 sec), in which participants were required to recall as much of the content of the sentence as they could during the time allowed. In the sparse fMRI design, a 1-sec period of imaging data acquisition was conducted following each trial. This sequence of trials was repeated for each sentence (Fig. 1). The verbal memory task consisted of 96 sentences across 2 scanning sessions. Among these, there were 32 sentences following no number trials (R0, 14 sec for each sequence), 32 sentences following 1 number trial (R1, 21 sec for each sequence), and 32 sentences following 2 number trials (R2, 28 sec for each sequence). Thus in total, the verbal memory task inside the fMRI scanner lasted 33 min 36 sec. For each sentence, the spoken responses were recorded and scored according to the percentage of content words retrieved.

### Imaging data acquisition and preprocessing

MRI data were acquired on a Siemens MAGNETOM Prisma 3T scanner (Siemens, Erlangen, Germany) with a 20-channel phased array head coil. Tight but comfortable foam padding was used to minimize head movement, and earplugs were used to reduce scanner noise. The functional images were collected using a T2*-weighted, gradient-echo, echo-planar imaging (EPI) sequence (repetition time = 1000 msec, echo time = 34.0 msec, flip angle = 62°, field of view = 230 × 230 mm^2^, slice number = 52, slice thickness = 2.50 mm). A high-resolution anatomical scan was also acquired for each subject using a T1-weighted, three-dimensional, magnetization-prepared, rapid gradient-echo sequence (repetition time = 2300 msec, echo time = 2.32 msec, inversion time = 900 msec, flip angle = 8°, field of view = 240 × 240 mm^2^, matrix size = 256 × 256, slice number = 192, slice thickness = 0.90 mm).

Image preprocessing was conducted with SPM12 software. The imaging data were slice-time corrected (to the middle slice), realigned to the first volume using a six-parameter rigid body transformation to correct for head motion. The high-resolution T1-structural image was segmented for gray/white matter and CSF. Then the parameters obtained from this step were subsequently applied to the functional (resampled to 3 × 3 × 3 mm^3^) data during normalization to Montreal Neurological Institute (MNI) space. The normalized images were further smoothed with a 6-mm full-width half-maximum Gaussian kernel.

### Behavioral and imaging analyses

For each participant, behavioral performance of the verbal memory task was assessed by the mean percentage of content words retrieved in each sentence. Given that an increased effort for memory retrieval is needed when the period of distraction between encoding and retrieval is lengthened (Dhanjal et al., 2013), we also calculated memory retrieval performance for R0, R1, and R2 separately. Then difference scores were calculated by subtracting the performance in the R2 condition from that in the R0 condition. The size of the difference scores could be regarded as a measure of effortful memory retrieval. A higher difference score was indicative of greater effortful memory retrieval.

For the imaging data, the general linear modeling was used in the first-level analysis to obtain contrasts of interest for each subject, with six head motion parameters accounting for residual movement artifacts. Because we were specifically interested in the neural correlates of memory encoding, the contrasts of interest only involved memory encoding activation (memory encoding versus number baseline). One sample *t* test was used to examine the general brain activation pattern during memory encoding for each group separately. Then the two-sample *t* test was used to identify brain activation with significant group differences.

Statistical significance for the brain activation within the standard automated anatomical labeling mask (47,636 voxels) was defined using the Analysis of Functional Neuroimages (AFNI) software's updated 3dClustSim program (Cox et al., 2017). The 3dClustSim program can conduct a 10,000 iteration Monte Carlo simulation of random noise activations at a particular voxel-wise α level within a masked brain volume. These simulations determined that a cluster-level corrected threshold of *p* < 0.05 was achieved using a voxel-wise threshold of *p* < 0.001 combined with a minimal cluster size of 30 voxels. Mean beta values were extracted from each of the clusters showing significant activation differences between the two groups. Then we conducted correlational analyses to examine whether altered brain activation during memory encoding was significantly correlated with subsequent memory retrieval performance. Specifically, partial correlational analyses were conducted while including age and gender as covariates. Bonferroni correction was applied for multiple comparisons.

## Results

### Behavioral performance

An independent samples *t*-test showed that the patients performed worse on the overall memory retrieval performance than the controls [*t*(35) = 3.179, *p* = 0.003]. Then, repeated-measures analysis of variance was carried out, including Group (patients or HCs) as a between-subject factor and Condition (R0, R1, or R2) as a within-subject factor. The results showed significant main effects of both Group [*F*(1, 35) = 9.939, *p* = 0.003] and Condition [*F*(2, 70) = 71.737, *p* < 0.001], as well as a significant interaction between Group and Condition [*F*(2, 70) = 6.594, *p* = 0.002]. *Post hoc* analysis indicated worse memory retrieval performance for the patients than the controls during all the three conditions [R0: *t*(35) = 2.911, *p* < 0.0065; R1: *t*(35) = 3.017, *p* = 0.005; R2: *t*(35) = 3.200, *p* = 0.003].

**FIG. 2.**
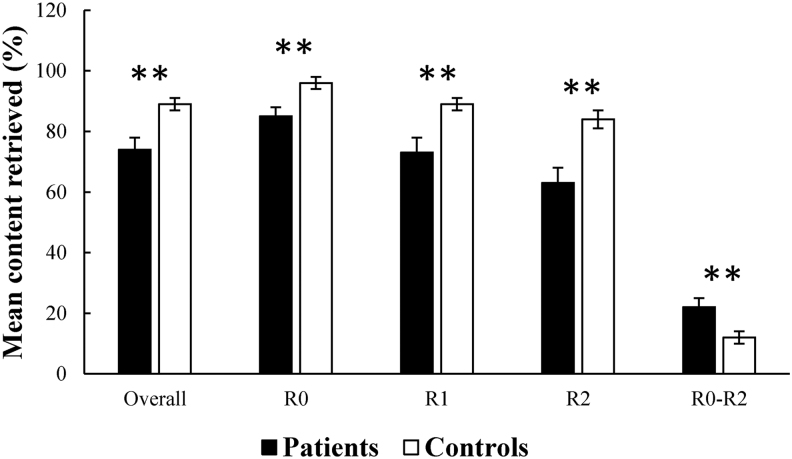
Behavioral performance for both groups. The patients showed significant memory retrieval impairment than the controls. R0, 0 number. R1, 5 numbers. R2, 10 numbers. R0–R2, difference scores between R0 and R2. Error bar shows one standard error of the mean. **p < 0.01.

*Post hoc* analysis also showed significant differences in memory retrieval between the three conditions [R0 versus R1: *t*(38) = 6.808, *p* < 0.05; R1 versus R2: *t*(38) = 8.514, *p* < 0.05; R0 versus R3: *t*(38) = 7.128, *p* < 0.05]. Thus, we calculated difference scores between R0 and R2 to assess effortful memory retrieval. Interestingly, the patients showed significantly larger difference scores than the controls [*t*(35) = 2.836, *p* = 0.008], indicating greater effortful retrieval for the patients. Figure 2 illustrates the behavioral performance for both groups.

### Brain activation

**FIG. 3.**
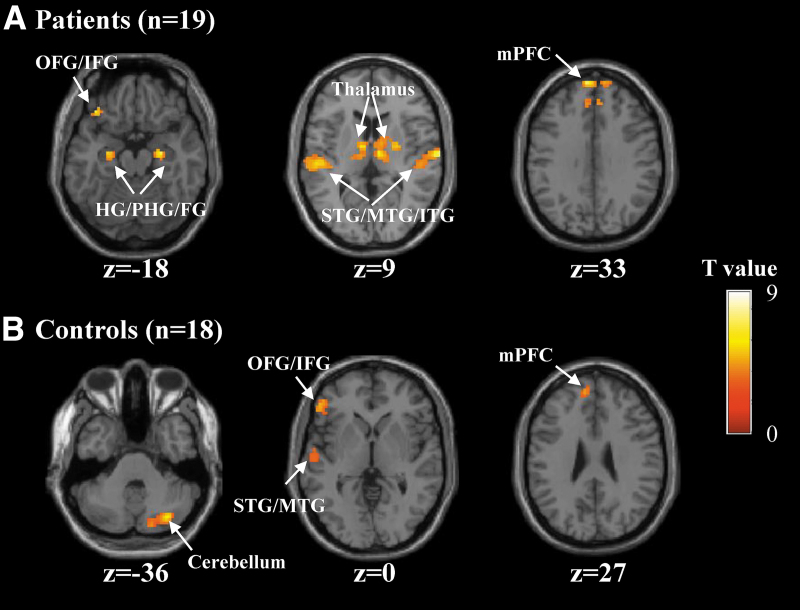
Brain regions showed significant activation during the memory encoding versus number baseline comparison. **(A)** Activation in the patients and **(B)** activation in the healthy controls. All images were thresholded at voxel-wise *p* < 0.001 and a minimal cluster size of 30 voxels. The left side of each axial slice corresponds to the left hemisphere of the brain. FG, fusiform gyrus; HG, hippocampus; IFG, inferior frontal gyrus; ITG, inferior temporal gyrus; mPFC, medial prefrontal cortex; MTG, middle temporal gyrus; PHG, parahippocampus; STG, superior temporal gyrus. Color images are available online.

An initial comparison between all encoding trials and the odd/even number trials was performed (Table 2). In the patients, the memory encoding condition exhibited bilateral activation in the HG/parahippocampus (PHG), fusiform gyrus (FG), inferior/middle/superior temporal gyrus (ITG/MTG/STG), medial prefrontal cortex (mPFC), and thalamus (Fig. 3A). There was also left-sided brain activation in the orbitofrontal/inferior frontal gyrus (OFG/IFG). In the HCs, the memory encoding condition induced activation in the left MTG/STG, mPFC, left OFG/IFG, and cerebellum (Fig. 3B). The brain regions reported above have been consistently reported to be activated in episodic memory encoding (Moscovitch et al., 2016).

**Table 2.** Summary of Significant Activation During the Memory Encoding Versus Number Baseline Comparison for Each Group

Anatomical region	Hemisphere	MNI coordinates	Cluster size	Peak* t *value
Patients				
HG/PHG/FG	Left	(−36, −15, −27)	111	6.87
HG/PHG	Right	(27, −12, −18)	71	6.65
OFG/IFG	Left	(−39, 30, −15)	48	7.15
STG/MTG/ITG	Left	(−66, −15, 0)	254	9.04
STG/MTG/ITG	Right	(69, −12, 6)	202	9.95
Thalamus	Bilateral	(9, −12, 9)	166	6.03
mFPC	Left	(−9, 63, 9)	40	6.19
mPFC	Bilateral	(−6, 60, 33)	62	6.99
Controls				
STG/MTG	Left	(−48, −9, −6)	91	4.97
OFG/IFG	Left	(−51, 33, 3)	54	4.72
mPFC	Left	(−9, 51, 27)	62	4.37
Cerebellum	Right	(33, −75, −36)	96	5.77

FG, fusiform gyrus; HG, hippocampus; ITG, inferior temporal gyrus; MNI, Montreal Neurological Institute; mPFC, medial prefrontal cortex; MTG, middle temporal gyrus; OFG/IFG, orbitofrontal/inferior frontal gyrus; PHG, parahippocampus; STG, superior temporal gyrus.

**FIG. 4.**
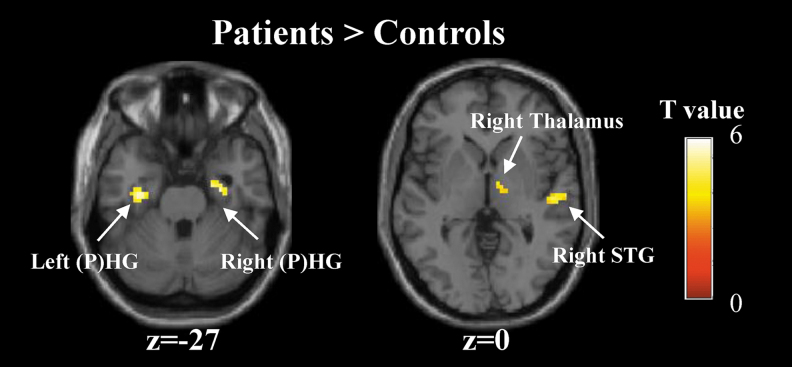
Group differences in brain activation during the memory encoding versus number baseline contrast. Four clusters that are significant for the two-sample *t* test. Color images are available online.

Then brain activation with significant group differences was examined using the two-sample *t* test. This detected four significant clusters, including the left HG/PHG, right HG/PHG, right STG, and right thalamus (Fig. 4 and Table 3). Among all of the four clusters, the patients showed greater activation than the controls during the period of verbal memory encoding (Fig. 4). However, no regions showed significantly greater activation in the HCs than the patients.

**Table 3.** Brain Regions Showed Greater Activation in the Patients than the Healthy Controls

Anatomical region	Hemisphere	MNI coordinates	Cluster size	Peak* t *value
HG/PHG	Left	(−33, −15, −27)	57	5.42
HG/PHG	Right	(30, −12, −27)	32	5.14
STG	Right	(57, −18, −3)	54	5.40
Thalamus	Right	(9, −6, 3)	34	4.73

**FIG. 5.**
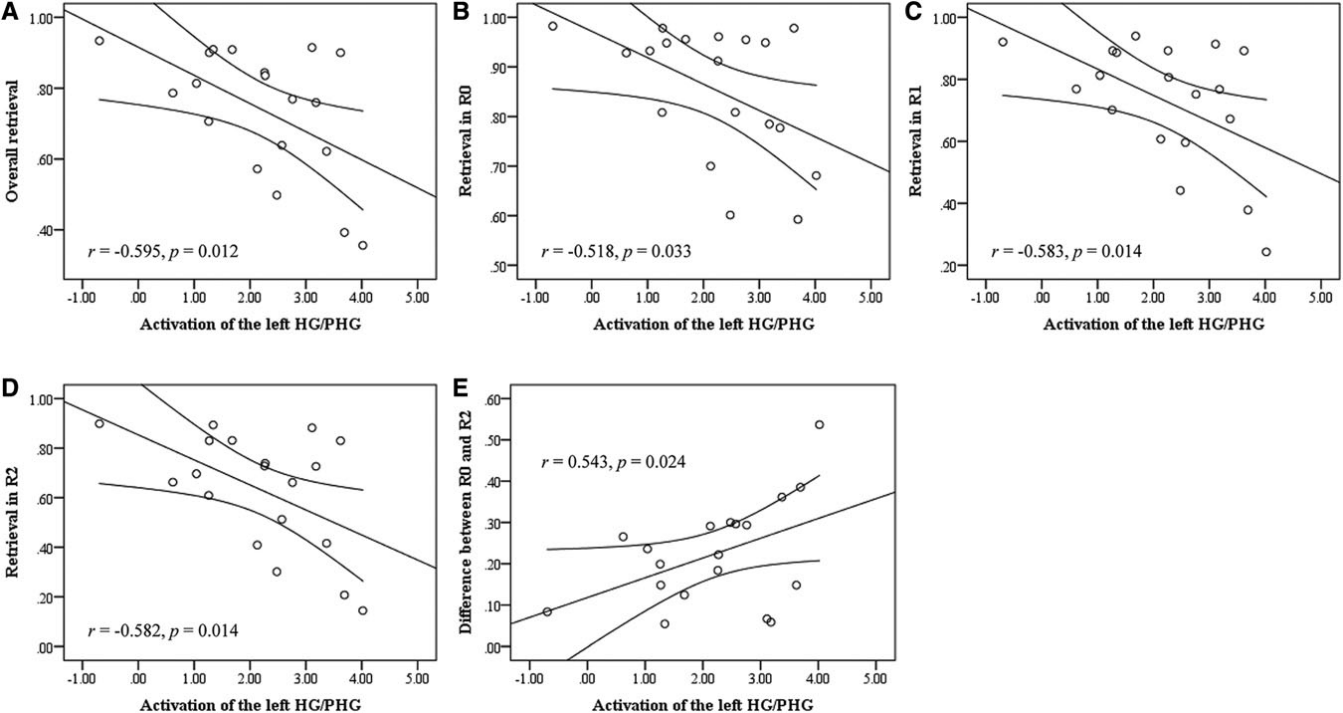
Brain–behavior correlations. **(A)** A negative correlation was found between activation of the left HG/PHG and overall content retrieval performance in the patients. **(B–D)** Similar correlations were found for the R0, R1, and R2 conditions. **(E)** A positive correlation was found between activation of the left HG/PHG and retrieval difference between the R0 and R2 conditions in the patients.

Among the patients, brain–behavior correlations were only found for the left HG/PHG. When controlling for age and gender in a partial correlation, greater encoding-related brain activation in the left HG/PHG was significantly correlated with worse overall memory retrieval performance (*r* = −0.595, *p* = 0.012, Fig. 5A). This correlation could survive Bonferroni correction at 0.05 (corrected to α = 0.0125 for four seed regions). Further analyses showed that this correlation reached significance for all three task conditions (R0: *r* = −0.518, *p* = 0.033; R1: *r* = −0.583, *p* = 0.014; R2: *r* = −0.582, *p* = 0.014, Fig. 5B–D).

Moreover, greater encoding-related brain activation in the left HG/PHG was correlated with larger memory retrieval difference between R0 and R2 (*r* = 0.543, *p* = 0.024, Fig. 5E), although the correlation did not survive Bonferroni correction for the number of correlations per region of interest. There were no brain–behavior correlations for the HCs (lowest *p* = 0.085). In addition, given that our data possessed fMRI data with two scanning sessions, we analyzed each session separately to explore the intersession reliability. The results were similar (Supplementary Data).

## Discussion

The current study sought to address whether the stage of memory encoding is compromised in patients with postacute anti-NMDA receptor encephalitis and how the effects of postacute anti-NMDA receptor encephalitis on memory encoding are substantiated at the neural level. Thus, we examined memory performance and associated brain activations in participants with and without a history of anti-NMDA receptor encephalitis via event-related fMRI during memory encoding. Consistent with previous studies (Finke et al., 2012; McKeon et al., 2018), we found that participants with postacute anti-NMDA receptor encephalitis showed worse memory retrieval performance than the HCs. In addition, the patients showed a significantly larger performance difference between conditions with and without numerical interference factors, indicating that the patients had a greater memory effortful retrieval when interference was present. Notably, a novel extension of previous work is the findings that the patients exhibited greater brain activation than the HCs during memory encoding. These activation differences during encoding may help explain why patients with postacute anti-NMDA receptor encephalitis recalled fewer items than the HCs did in our study and in previous studies (Finke et al., 2012; McKeon et al., 2018). Taken together, the present findings add specificity to previous results and shed some light on the role of NMDA receptors in human memory encoding.

Whole-brain analysis of brain activation revealed greater activation for memory encoding versus number baseline in the bilateral HG/PHG (Table 2 and Fig. 3). This is consistent with previous findings that the HG/PHG is critical for mediating memory encoding (Moscovitch et al., 2016). It has been suggested that the HG/PHG sits at the top of a hierarchy of largely cortical systems (Nadel and Peterson, 2013), and obligatorily binds neocortical structures together into a memory trace or engram that gives rise to the multimodal, multidomain representations during encoding (Dudai, 2012; Josselyn et al., 2015; Tonegawa et al., 2015). In addition, whole-brain analyses revealed that, during encoding, greater brain activation was observed in the bilateral FG, ITG/MTG/STG, mPFC and left precuneus, MFG, OFG/IFG (Table 2 and Fig. 3). These brain regions have been also previously reported to engage during memory encoding (Dickerson et al., 2007; Moscovitch et al., 2016). Moreover, functional interactions of these brain regions with the HG/PHG have been suggested to make memory intelligent and goal-directed behavior (Moscovitch et al., 2016).

It is important to highlight that the effect of anti-NMDA receptor encephalitis on memory was found during encoding. The patients showed greater activation in several regions compared with the HCs. The dynamic nature of abnormalities in patients suggests a functional disruption, despite substantial recovery after timely immunotherapy in patients with anti-NMDA receptor encephalitis. Specifically, the patients overactivated the bilateral HG/PHG during the period of verbal memory encoding. This finding extended the results of several previous studies reporting reduced hippocampal FC, hippocampal volumetrics, and microstructural integrity and relationships with memory performance in patients with postacute anti-NMDA receptor encephalitis (Finke et al., 2013, 2016; Peer et al., 2017; Wang et al., 2019). Taken together, these findings may suggest that anti-NMDA receptor encephalitis places the HG at risk for vulnerability both in terms of structure and function, which may lead to individual memory impairments.

Another novel finding of this study was a significantly higher activation in the right STG and right thalamus during memory encoding in the patients than the HCs. The maintenance of auditory attention and semantic processing, which are mediated in the lateral temporal cortex located at the auditory cortex, are important to episodic memory encoding (Moscovitch et al., 2016). It was found that impaired registration of verbal messages, adding to an impairment consequent upon poor encoding, may be associated with altered auditory cortical function in Alzheimer's disease (Dhanjal et al., 2013). Consistent with previous studies, altered activation in the right STG located at the auditory cortex was found during auditory verbal memory encoding in patients with anti-NMDA receptor encephalitis. For the thalamus, its importance in human memory is becoming indubitable, due to the strong connection to other regions (Aggleton et al., 2010; Oh et al., 2012). Overactivation in these regions may reflect inefficient, compensatory brain activity, as extraneous task-related activation can also be observed at the early stages of learning before an optimal cognitive strategy has been reached (Ramsey et al., 2004), and during reorganization following acute brain injury (Johansen-Berg, 2007).

Furthermore, among the patients, a significant negative correlation in all task conditions was observed between memory retrieval performance and encoding-related activation within the left HG, implying that poor memory retrieval performance in patients is likely attributed to dysfunction of left HG during verbal memory encoding. Interestingly, we also found that greater encoding-related brain activation in the left HG was correlated with greater effortful memory retrieval. Combined with behavioral results, our study showed that not only memory impairments but also a greater sensitivity to the effects of distraction may be related to the altered modulation of HG function in patients with postacute anti-NMDA receptor encephalitis past the acute stage.

This is the first study, to our knowledge, to utilize task-based fMRI to investigate the effects of postacute anti-NMDA receptor encephalitis on neural correlates of memory encoding. However, a few limitations should be noted. First, the present study focused on patients after the acute stage (at least 6 months after initial discharge from the hospital). It remains unclear how the memory encoding pattern is influenced by selective NMDA receptor disruption at an earlier stage. Second, the patients in this study received multiple treatments such as immunosuppressants, antiepileptic drugs at the acute stage. These treatments might bring irreversible cognitive impairment or have residual effects on cognition even after treatment discontinuation. Thus, group differences in our findings might be partly driven by these treatments other than the dysfunctional NMDA receptor only. Further longitudinal studies from more stages are needed to gain a deeper insight into the neural mechanisms underlying memory deficits in anti-NMDA receptor encephalitis.

## Conclusion

In conclusion, results from the present study suggest that brain dysfunctional activation during the memory encoding process may affect the subsequent memory retrieval performance in patients with postacute anti-NMDA receptor encephalitis. Overactivation in the bilateral HG/PHG, right STG, and thalamus at the period of encoding suggests that the stage of memory encoding is compromised in patients with postacute anti-NMDA receptor encephalitis, and greater brain activity in these brain regions may reflect inefficient or compensatory encoding processes. Furthermore, variations in HG activation during encoding were significantly correlated with subsequent memory retrieval performance. These findings not only provide a new perspective to the neural mechanism underlying memory deficits in patients with postacute anti-NMDA receptor encephalitis, but also imply that interventions designed to improve the memory ability of such patients should give a special focus on the memory encoding process. With anti-NMDA receptor encephalitis providing a unique human model of selective NMDA receptor disruption, these observations may enhance our understanding of NMDA receptor dysfunction in the human brain during verbal memory encoding.

## Supplementary Material

Supplemental data
