# Supplementary material for: Abnormal Brain Activation During Verbal Memory Encoding in Postacute Anti-N-Methyl-d-Aspartate Receptor Encephalitis
Source: Brain Connect. 2022 Sep 15;12(7):660–9. doi: 10.1089/brain.2021.0046 (PMC9527060; doi:10.1089/brain.2021.0046)
Supplement: Supplemental data [file Supp_DataS1.docx]

Given that our data possessed fMRI data with two scanning sessions, we decided to analyze each session separately to explore the inter-session reliability of the present work. Specifically, we attempted to replicate within each session of time series, the two important findings of this study, i.e. (1) group differences in encoding-related brain activation; and (2) correlations between encoding-related brain activation and memory retrieval performance. Interestingly, the two important findings mentioned above could still be replicated overall.

First, the four clusters showing significant group differences in memory-related brain activation in the original manuscript were used as regions of interest. The mean beta values were extracted from each cluster. Interestingly, for each session, activation of each cluster in the patients was significantly higher than that of the controls (rFigure 1).


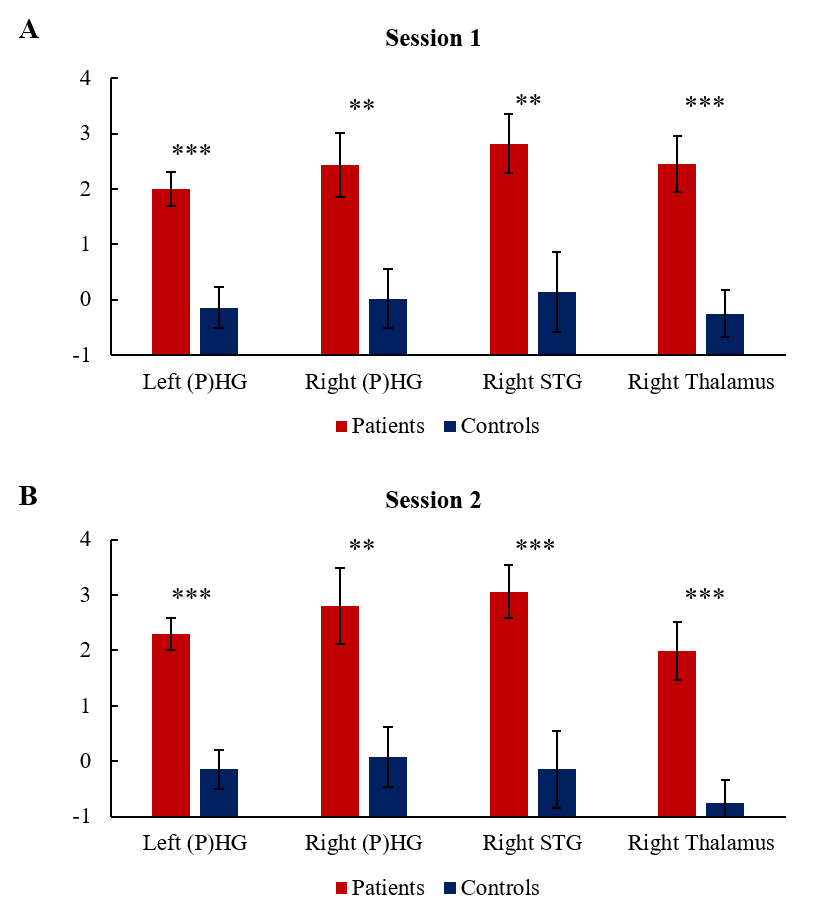


**rFigure 1 Validation analyses for group difference in encoding-related activation during each session.** STG, superior temporal gyrus; HG, hippocampus; PHG, parahippocampus. ^***^ *p* <0.001; ^**^ *p* < 0.01.

Second, we examined correlations between memory-related activation and memory retrieval performance. Importantly, for the first session, greater encoding-related activation of the left HG/PHG among the patients was associated with worse memory retrieval performance in all task conditions while controlling for age and gender (Overall: *r* = -0.555, *p* = 0.021; R0: *r* = -0.517, *p* = 0.034; R1: *r* = -0.536, *p* = 0.027; R2: *r* = -0.537, *p* = 0.026, rfigure 2A-D). There was also a marginal correlation between activation of the left HG/PHG and effortful memory retrieval (R0-R2: *r* = 0.460, *p* = 0.063, rfigure 2E).


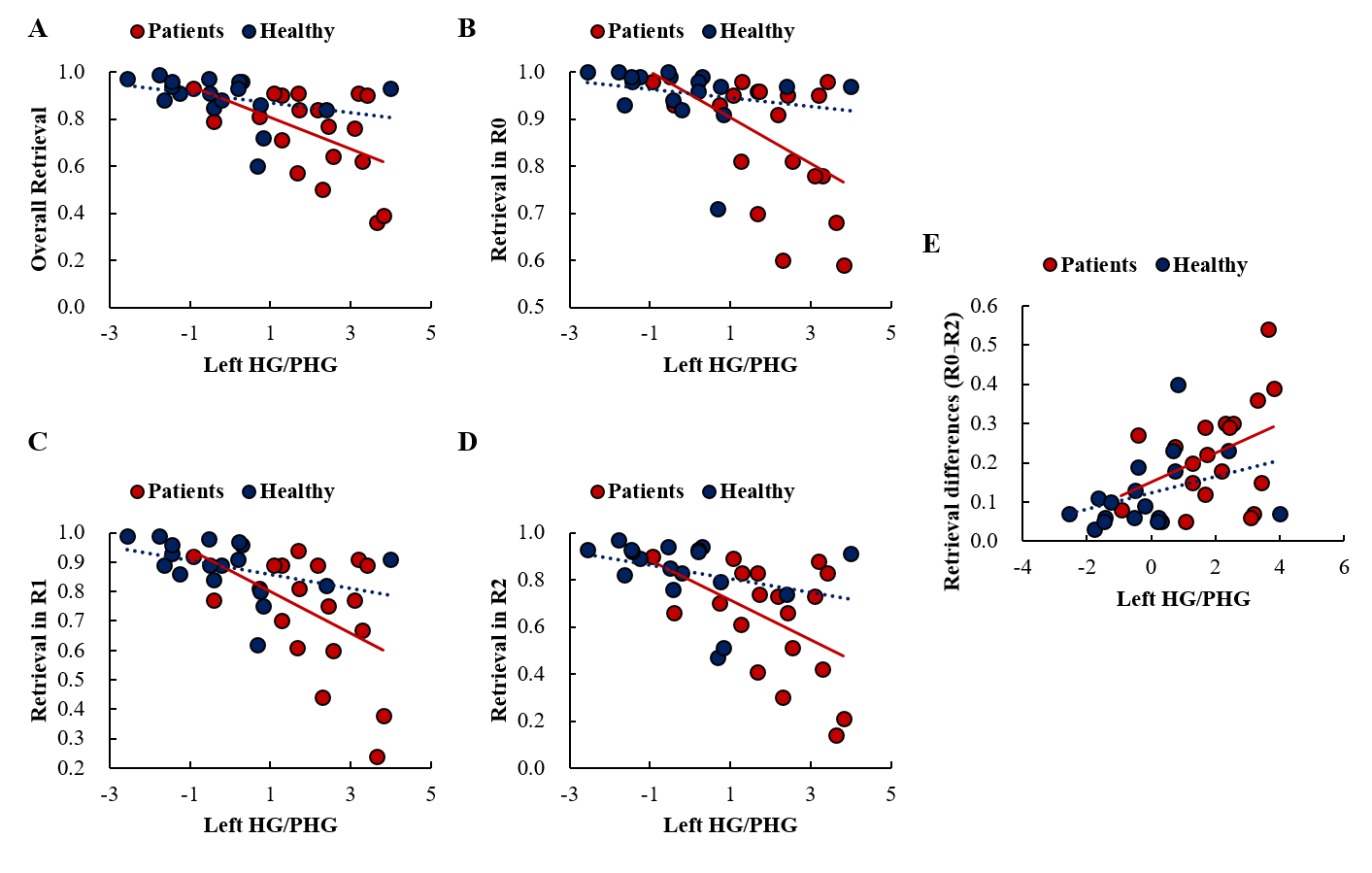


**rFigure 2 Brain-behavioral correlations for the first session.** (A) A negative correlation was found between activation of the left HG/PHG and overall content retrieval performance in the patients. (B-D) Similar correlations were found for the R0, R1 and R2 conditions. (E) A positive correlation was found between activation of the left HG/PHG and retrieval difference between R0 and R2 conditions in the patients. HG, hippocampus; PHG, parahippocampus.

Similar correlations were also found for the second session (Overall: *r* = -0.579, *p* = 0.015; R0: *r* = -0.480, *p* = 0.051; R1: *r* = -0.575, *p* = 0.016; R2: *r* = -0.578, *p* = 0.015: R0-R2: *r* = 0.575, *p* = 0.016, rfigure3).


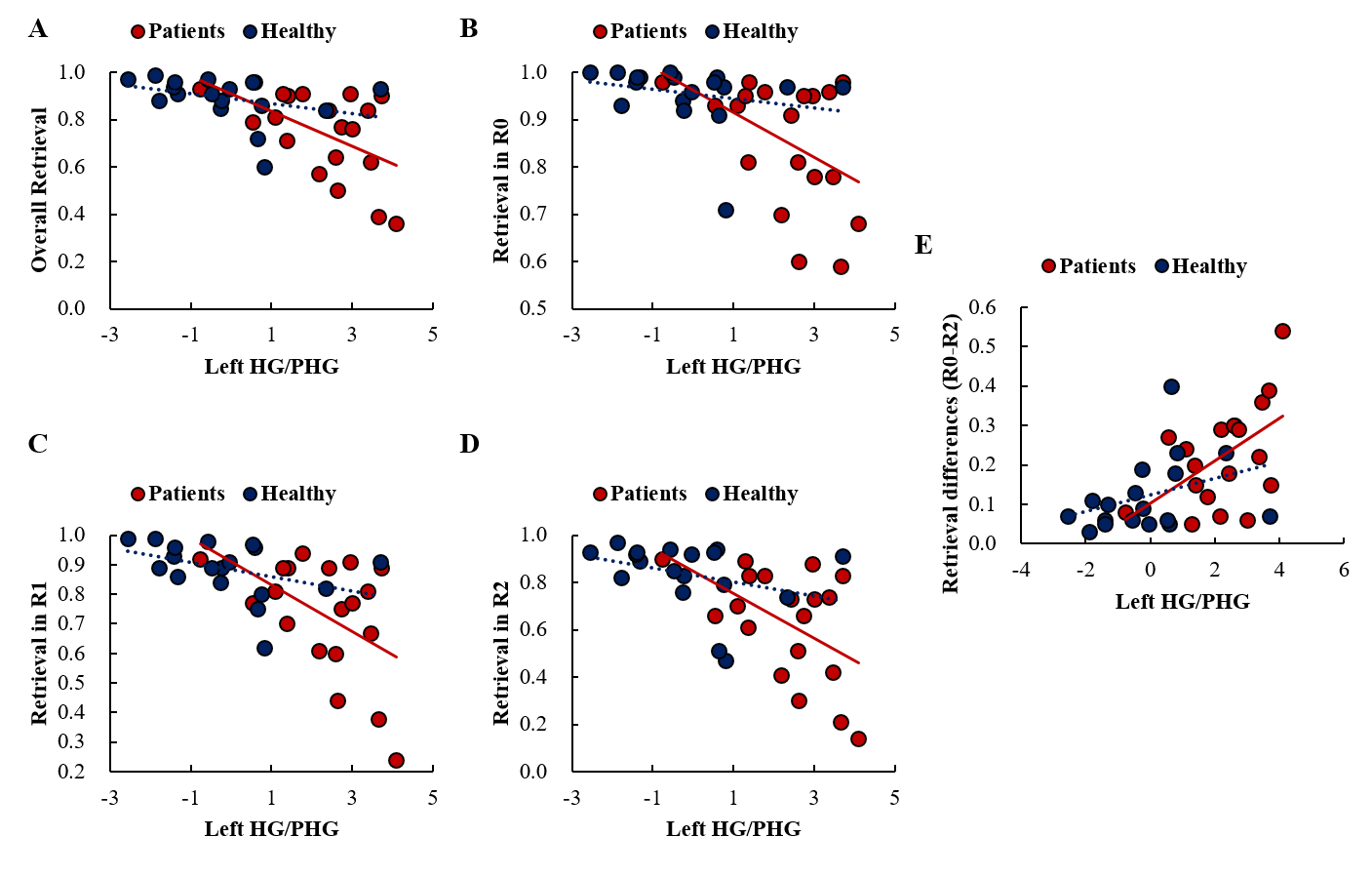


**rFigure 3 Brain-behavioral correlations for the second session.** (A) A negative correlation was found between activation of the left HG/PHG and overall content retrieval performance in the patients. (B-D) Similar correlations were found for the R0, R1 and R2 conditions. (E) A positive correlation was found between activation of the left HG/PHG and retrieval difference between R0 and R2 conditions in the patients. HG, hippocampus; PHG, parahippocampus.
